# Functions of Tomato (*Solanum lycopersicum* L.) Signal Transducer and Activator of Transcription (STAT) in Seed Germination and Low-Temperature Stress Response

**DOI:** 10.3390/ijms26073338

**Published:** 2025-04-03

**Authors:** Yidan Zhang, Jiahui Zhao, Jingyuan Li, Yanting Li, Libo Jiang, Na Wang

**Affiliations:** College of Life Sciences and Medicine, Shandong University of Technology, Zibo 255000, China

**Keywords:** tomato, STAT, seed germination, low temperature, m^6^A

## Abstract

Tomato (*Solanum lycopersicum* L.) is one of the major vegetable crops worldwide. Research on the Janus kinase–signal transducer and activator of transcription (JAK–STAT) signaling pathway in tomatoes and other plant systems is extremely limited. In this study, the roles of STAT, a crucial element of the JAK–STAT signaling pathway in tomato seed germination and low-temperature stress responses are examined, employing gene family analysis and genetic transformation. The results indicate that the *S. lycopersicum* genome contains only one member of the STAT gene family, *SlSTAT*. Subcellular localization experiments reveal that SlSTAT is found in both the cytoplasm and nucleus, suggesting its potential involvement in biological functions within these cellular compartments. Among the 26 different tomato tissue/organs tested, *SlSTAT* exhibited higher expression levels in hypocotyl (8 days past germination; 8 DPG), and low expression of *SlSTAT* significantly reduced the germination rate and impacted biomass at 8 DPG. In addition, the *SlSTAT* gene was significantly downregulated during low-temperature treatment. Compared with the wild-type (WT) tomatoes, the *SlSTAT*-overexpressing plants showed more resistance to low-temperature conditions, whereas the downexpressing tomatoes exhibited increased sensitivity. The expressions of low-temperature marker genes (*SlCBF1*-*3*) and *N*^6^-methyladenosine (m^6^A)-modification-related genes (m^6^A writer, reader, and eraser genes) were detected to explore possible molecular mechanisms by which *SlSTAT* causes changes in tomato low-temperature stress resistance. The expression changes of *SlCBF1*-*3* in transgenic plants do not merely follow a straightforward linear relationship with the changes in *SlSTAT* expression, suggesting a more complex molecular mechanism and a non-direct interaction between SlSTAT and the promoters of *SlCBFs*. On the other hand, *SlSTAT* also changes the expression levels of RNA m^6^A-modification-related genes, especially *SlFIP37* (writer gene), *SlYTP8/9* (reader genes), and *SlALKBH8* (eraser gene), ultimately leading to changes in the levels of m^6^A modification. These research findings lay the groundwork for exploring functions of JAK–STAT pathway in tomato development and stress responses, expanding the scope of JAK–STAT signaling studies in plant systems.

## 1. Introduction

As early as 1957, scientists discovered the existence of the Janus kinase–signal transducer and activator of transcription (JAK–STAT) pathway while studying how cells respond to interferons (IFNs) and gradually elucidated its structure and function [1]. Through continuous exploration, although there are still some unverified and unresolved challenges, the composition and signaling process of the JAK–STAT pathway have been thoroughly studied [2,3]. Typically, the JAK–STAT signaling pathway consists of four components: extracellular signals (cytokines), membrane receptors, intracellular JAKs, and STATs. After extracellular cytokines bind to receptor proteins on the cell membrane, they induce the dimerization or multimerization of the receptors. This promotes the association of JAK proteins with the intracellular domains of receptors, subsequently recruiting and phosphorylating STAT proteins [4]. After phosphorylation, the STAT proteins dissociate from the receptors and are transported from the cytoplasm to the nucleus. Furthermore, phosphorylation induces a conformational change in STAT proteins, which facilitates their binding to specific DNA sequences. Subsequently, STATs regulate gene expression by influencing the transcription process [5]. The seemingly simple JAK–STAT signaling pathway plays a crucial role in many important biological processes, such as cell proliferation, differentiation, migration, apoptosis, and immune system regulation [5,6].

STATs are involved in the final stage of JAK–STAT signaling transduction. The animal STAT family comprises seven members (STAT1, STAT2, STAT3, STAT4, STAT5A, STAT5B, and STAT6) [7]. Phosphorylated STATs translocate from the cytoplasm to the nucleus, where they directly bind to the promoters of target genes to regulate their expression levels [5]. In addition to binding to gene promoters individually, STATs can also interact with transcription regulatory proteins, including transcription factors, to exert their effects collaboratively [8,9]. Recently, Bhattarai et al. reported that METTL3-STAT5B interaction facilitates the co-transcriptional *N*^6^-methyladenosine (m^6^A) of messenger RNA (mRNA) to promote breast tumorigenesis [10]. The RNA m^6^A modification is one of the most common types of RNA modification in eukaryotes [11]. The cellular RNA m^6^A modification level is decided by m^6^A writer (methyltransferase) and eraser (demethylases) proteins and recognized by reader proteins [12]. In addition, an increasing number of researchers have focused on the interactions between STATs/JAK–STAT signaling pathway and RNA m^6^A modification [13]. However, reports on STAT in the field of plants are extremely limited. The existing literature largely focuses on studies examining the effects of traditional Chinese herbal medicine extracts (TCHMs) on the changes in the JAK–STAT signaling pathway in animal cells [14]. Research on the function of plant STATs is relatively scarce.

Tomato *(Solanum lycopersicum* L.) is one of the most widely cultivated vegetables globally and is also a frequently used model plant in scientific research. To meet the increasing demands of both domestic and international markets for higher tomato yield and quality, it is essential to start by studying the germination of tomato seeds. This involves identifying key factors that enhance seed germination rates and elucidating the underlying mechanisms to provide a theoretical and experimental foundation for breeding programs aimed at improving tomato yield and quality [15,16,17]. On the other hand, climate change has significantly intensified occurrences of extreme temperatures, thus threatening the sustainability of crop production worldwide [18]. This has led to low temperatures becoming one of the ecological constraints for outdoor and off-season tomato cultivation [19,20]. Exploring novel signaling pathways that differ from the conventional CBFs (C-repeat binding factors)-centred transcriptional regulatory network and analyzing the functions of new genes under low-temperature stress can provide new perspectives and directions for breeding tomatoes with low-temperature tolerance [21].

In this study, Micro-Tom tomatoes (*S. lycopersicum* cv. Micro-Tom) are used as experimental material to explore the characteristics of the *STAT* genes during the tomato seed germination period and their functions in response to low-temperature stress. The *SlSTAT* gene family members are identified in the tomato genome. After generating overexpression (OE) and RNA interference (RNAi) transgenic tomato constructions, phenotypic comparison experiments were conducted to investigate the impact of SlSTAT expression on tomato seed germination and cold resistance. Furthermore, the possible molecular mechanisms underlying phenotypic differences were preliminarily explored by measuring the expression levels of cold marker genes and m^6^A-related genes. This study can lay the groundwork for exploring the JAK–STAT pathway in plant cold stress responses and identifying its detailed relationship with the conventional cold response network or RNA m^6^A modification.

## 2. Results

### 2.1. SlSTAT Family Member Identification and Analysis

The protein sequences of the seven human identified *HsSTAT*s were acquired from the NCBI website (https://www.ncbi.nlm.nih.gov/; accessed on 30 March 2025) and used for detection of *AtSTAT*s and *SlSTAT*s through two iterations of BLASTP. A total of two and one STATs were identified by two round BLASTP in *A. thaliana* and *S. lycopersicum* genome, respectively (Table S1). The evolutionary relationships and classification of these STATs have been clarified through the construction of a phylogenetic tree using the protein sequences of 7 HsSTATs, 2 AtSTATs, and 1 SlSTAT. In comparison to animals, the number of STAT family members in Arabidopsis and tomato is relatively limited, and their phylogenetic relationship to human STATs is considerably more distanced (Figure S1).

**Figure 1:**
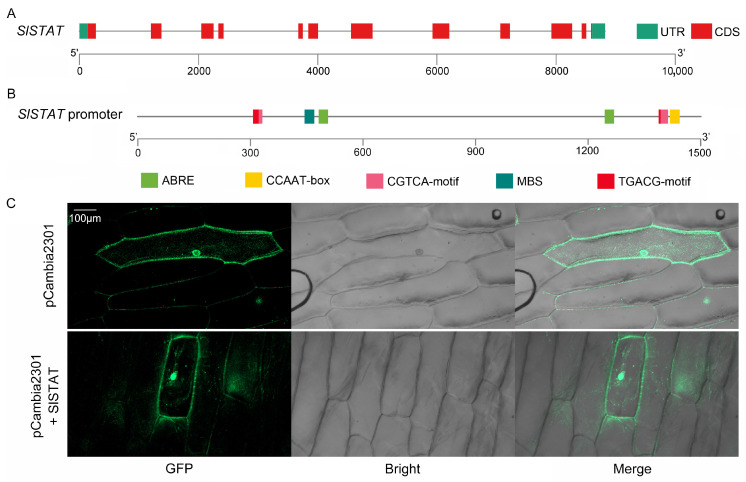
Gene structure, promoter analysis, and subcellular localization of *SlSTAT*. (**A**) Gene structure of *SlSTAT*, illustrating the coding sequence (CDS), untranslated regions (UTRs), and introns represented by lines connecting the boxes. (**B**) Analysis of *cis*-acting elements in the *SlSTAT* promoter, with various types of elements indicated by color-coded boxes. (**C**) Subcellular localization of SlSTAT, shown through confocal microscopy images of onion epidermal cells co-expressing either GFP alone (pCambia2301) or SlSTAT-GFP (pCambia2301 + SlSTAT). From left to right, the columns display GFP fluorescence, bright field, and merged images. The scale bar indicates a measurement of 100 μm.

After gene cloning, the *SlSTAT* (*Solyc07g062050*) CDS was obtained, with a length of 2112 bp (Table S2). Through alignment with the *SlSTAT* gene sequences containing introns in the tomato genome, we identified 12 exons and 11 introns within the *SlSTAT* genomic genes (Figure 1A). The 1500 bp sequence upstream of the *SlSTAT* gene was selected as the promoter region. Using genomic DNA as a template, the *SlSTAT* promoter was cloned, followed by the promoter *cis*-acting element predictions. The results indicated the presence of various stress-related elements, including abscisic acid responsiveness, MeJA responsiveness, and MYB-binding sites associated with drought inducibility, suggesting the gene’s involvement in stress and plant hormone response mechanisms (Table S3, Figure 1B). To investigate the subcellular localization of SlSTAT proteins, a fusion of SlSTAT with GFP was transiently expressed in onion epidermal cells. The results demonstrated that the SlSTAT protein was localized in both the cytoplasm and the nucleus (Figure 1C).

### 2.2. Expression Levels of SlSTAT in Different Tomato Tissue/Organs

To confirm the tissue-expression of the *SlSTAT* gene, its expressions in 26 different tomato tissues were obtained from the tomato transcription database of various tissue/organs (Table S4) [22]. Subsequently, its expression patterns are represented using the cartoon heatmap. Notably, the *SlSTAT* gene exhibits relatively low expression across various tomato tissues and organs, with FPKM values ranging from 1.22 (3 DPG seed) to 3.83 (8 DPG hypocotyl) (Table S4).

**Figure 2:**
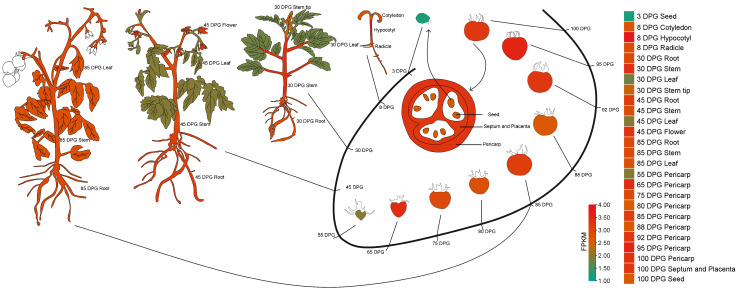
Expression patterns of *SlSTAT* in different tomato tissue/organs. The cartoon heatmap depicts *SlSTAT* gene expression patterns across various tomato tissues, with average expression values calculated from three independent biological replicates. The scale bar represents FPKM (Fragments Per Kilobase of transcript per Million mapped reads) values.

As illustrated in Figure 2, *SlSTAT* displays reduced expression levels in 3 DPG seed (early germination stage), 30 DPG leaf (vegetative growth stage), 45 DPG leaf (flowering stage), and 55 DPG pericarps (immature green fruiting stage). In contrast, *SlSTAT* exhibits elevated expression levels in 8 DPG hypocotyl (seedling stage). The increased expression from the early germination period to the seedling growth stage underscores the critical role of *SlSTAT* in seed germination processes.

### 2.3. Function of SlSTAT During Seed Germination Processes

**Figure 3:**
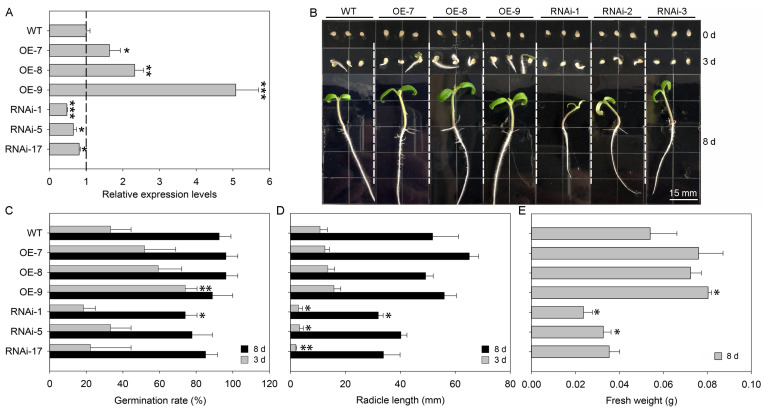
Expression of *SlSTAT* gene affects tomato seed germination and seedling growth. (**A**) Expression levels of *SlSTAT* in wild-type (WT), overexpression (OE), and RNA interference (RNAi) transgenic tomato plants. (**B**) Seed germination phenotype of WT, *SlSTAT* OE lines, and *SlSTAT* RNAi lines under 25 °C conditions. Scale bars = 15 mm. (**C**–**E**) Germination-related physiological indexes measurement, including germination rate (**C**), radicle length (**D**), and fresh weight (**E**). Error bars represent the Standard Deviation (SD). Asterisks denote statistical significance levels: *, **, and *** correspond to *p* <  0.05, *p* < 0.01, and *p* <  0.001, respectively.

To examine the impact of *SlSTAT* gene expression on tomato seed germination and seedling growth, we constructed OE and RNAi vectors for the *SlSTAT* gene. Subsequently, we conducted genetic transformation in tomato plants and confirmed the presence of transgenic plants at both the DNA and RNA levels (Figure S2 and Figure 3A). Ultimately, we established stable *SlSTAT* OE and RNAi transgenic tomato lines. For seed germination assays, the WT and transgenic tomato plant seeds were incubated at 25 °C for 8 days. Phenotypic observations were recorded at 0, 3, and 8 d (Figure 3B).

On day 3 of seed germination, only the OE-9 line exhibited a significantly higher germination rate compared to the WT tomatoes (Figure 3C). No significant differences were observed among the other OE lines, RNAi lines, and WT plants (Figure 3C). However, the radicle lengths of all RNAi lines were significantly shorter than those of the WT tomatoes (Figure 3D). In contrast, no significant differences in radicle lengths were found between the OE lines and WT plants (Figure 3D).

On day 8 of germination, only the RNAi-5 line demonstrated a significantly lower germination rate and radicle length compared to the WT plants. The other OE and RNAi lines exhibited no significant differences in either germination rate or radicle length relative to the WT tomatoes (Figure 3C,D). However, the fresh weight of the OE-9 line was significantly higher than that of the WT. In contrast, the fresh weights of the RNAi-1 and RNAi-5 lines were significantly lower than those of the WT (Figure 3E). The experimental findings demonstrate that *SlSTAT* expression significantly affects the germination of tomato seeds, particularly in those with reduced *SlSTAT* expression levels (RNAi lines). Early in the germination process, decreased *SlSTAT* expression notably reduces the radicle lengths and negatively impacts biomass.

### 2.4. Expression of SlSTAT Change the Chilling Resistance of Tomato

**Figure 4:**
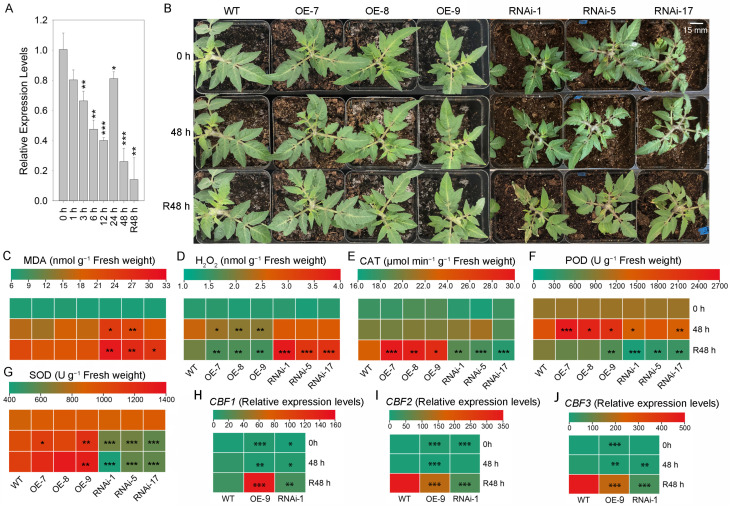
The relationship between *SlSTAT* expressions and tomato resistance under low-temperatures stress. (**A**) The relative expression levels of *SlSTAT* in tomato leaves subjected to low-temperature stress were assessed. The expression levels at other time points were calculated relative to the 0 h. (**B**) Phenotype of WT tomatoes, *SlSTAT* OE lines, and *SlSTAT* RNAi lines under low-temperature conditions. Scale bars = 15 mm. (**C**–**G**) Stress-related physiological indexes measurement, including malondialdehyde (MDA) concentration (**C**), hydrogen peroxide (H_2_O_2_) concentration (**D**), catalase (CAT) activity (**E**), peroxidase (POD) activity (**F**), and superoxide dismutase (SOD) activity (**G**). (**H**–**J**) Relative expression levels of cold stress marker genes, including *SlCBF1* (**H**), *SlCBF2* (**I**), and *SlCBF3* (**J**), in WT and transgenic tomato leaves under low-temperature stress treatment. The mean expression levels were calculated in comparison to the WT plants at time point 0 h. Significance analyses were separately conducted at 0 h, 48 h, and R48 h, with all significant difference comparisons being made against the WT tomatoes. Asterisks denote statistical significance levels: *, **, and *** indicate *p* <  0.05, *p* < 0.01, and *p* <  0.001, respectively.

Low temperatures severely affect the yield and quality of tomatoes. The expression of the *SlSTAT* gene is significantly downregulated during cold treatment, with the lowest expression observed after 48 h at low temperatures. Even after returning to normal growth conditions (R48 h), the expression of *SlSTAT* does not recover (Figure 4A). To explore the specific role of *SlSTAT* in the response to low-temperature stress, we analyzed the phenotype and physiological parameters of OE and RNA interference (RNAi) lines in comparison to wild-type (WT) plants following low-temperature treatment.

From a phenotypic perspective, there are no significant differences between the *SlSTAT* OE plants and the WT tomato plants, whether under 25 °C or 4 °C growth conditions (Figure 4B). Moreover, in accordance with the phenotypic findings, there were no significant differences in the MDA content between the *SlSTAT* OE lines and WT tomato leaves under both normal temperature and low-temperature treatment conditions (Figure 4C). However, as illustrated in Figure 4D, at 48 h and R48 h, the leaves of *SlSTAT* OE tomatoes contained lower levels of H_2_O_2_ compared to the WT plants, showing a milder degree of cold damage in OE tomatoes. CAT, POD, and SOD are key enzymes in cells that eliminate reactive oxygen species. While no significant difference in CAT activity was observed between OE plants and WT plants at 48 h, the CAT activity was significantly elevated in the OE plants compared to the WT plants at R48 h (Figure 4E). For POD enzyme activities, all *SlSTAT* OE transgenic lines exhibited significantly higher levels than those in the WT plants at 48 h, and only the OE-9 line showed significantly higher POD enzyme activities at R48 h compared to the WT, which may be related to the higher gene expression of *SlSTAT* (Figure 3A and Figure 4F). Moreover, SOD enzyme activities in the OE-9 transgenic plant lines also showed higher levels than those in WT tomato plants at both 48 and R48 h (Figure 4G). We did not find significant differences in the H_2_O_2_ content and activity of the CAT\POD\SOD between OE and WT tomatoes at 0 h (Figure 4D–G). Thus, overexpression of *SlSTAT* enhanced the reactive oxygen species scavenging ability of tomato plants under low-temperature stress conditions. In summary, by decreasing peroxide accumulation in the leaves and increasing the activity of enzymes associated with reactive oxygen species scavenging, we obtained overexpression of *SlSTAT*-enhanced tomato cold stress resistance, which is positively correlated with *SlSTAT* overexpression levels.

Conversely, *SlSTAT* RNAi plants exhibited stunted growth compared to the WT plants at the same growth stage, and they demonstrated greater leaf curling in response to chilling stress, demonstrating increased susceptibility to low-temperature stress compared to the WT tomatoes (Figure 4B). In line with the phenotypic observations, no significant variance was observed in the MDA levels between *SlSTAT* RNAi and WT tomato leaves under 25 °C temperature conditions. However, following low-temperature treatment, the MDA content in the leaves of *SlSTAT* RNAi plants was significantly elevated compared to WT tomatoes at both 48 h and R48 h (Figure 4C). Similarly, the accumulation of H_2_O_2_ in the leaves of RNAi plants was greater than that observed in WT plants, particularly at the R48 h (Figure 4D). While no significant difference in CAT activity was observed between OE plants and WT plants at 48 h, the CAT activity in the RNAi plants was significantly lower than in WT plants at R48 h (Figure 4E). For POD enzyme activity, all *SlSTAT* RNAi transgenic lines exhibited significantly lower levels than those in the WT plants at R48 h, and only the RNAi-1 and RNAi-17 lines showed significantly lower POD enzyme activities at 48 h compared to the WT plants (Figure 4F). Moreover, SOD enzyme activity in all RNAi transgenic plant lines showed lower levels that those in the WT tomato plants at both 48 and R48 h (Figure 4G). Thus, downexpression of *SlSTAT* reduced the reactive oxygen species scavenging ability of tomato plants under low-temperature stress conditions. In summary, by increasing peroxide accumulation in the leaves and decreasing the activity of enzymes associated with reactive oxygen species scavenging, the downexpression of *SlSTAT* enhanced sensitivity to cold stress in tomato.

Meanwhile, the expression levels of the low-temperature response marker genes, *SlCBF1*-*3*, were assessed (Figure 4H–J, Table S5). Low temperatures increase the expression of these three genes, with *SlCBF2* and *SlCBF3* showing a particularly significant rise in expression at R48 h (Figure 4H–J). On one hand, the overexpression or underexpression of *SlSTAT* affects the expression of *SlCBF* genes under normal growth conditions. Compared to the WT plants, the expression level of *SlCBF1* is up-regulated in OE lines and down-regulated in RNAi transgenic plants (Figure 4H). Interestingly, in contrast to the WT tomatoes, *SlCBF2* and *SlCBF3* are upregulated in both OE lines and RNAi transgenic plants, although the degree of up-regulation in the OE lines is higher than that in the RNAi transgenic plants (Figure 4I,J). On the other hand, the overexpression or downregulation of *SlSTAT* also affects the expression of these three genes after low-temperature treatment and during the recovery period of plant growth, particularly at R48 h (Figure 4H–J). At R48 h, the expression levels of *SlCBF1* in both the OE and RNAi plants were significantly higher than in the WT plant, with the expression in the OE lines also exceeding that in the RNAi plants (Figure 4H). Conversely, the expression levels of *SlCBF2* and *SlCBF3* in both the OE and RNAi lines were significantly lower than those in the WT plant, though the expression in the OE lines was still higher than that in the RNAi lines (Figure 4I,J). These results indicate that the expression changes of *SlCBF1*-*3* in transgenic plants do not merely follow a straightforward linear relationship with the changes in *SlSTAT* expression, suggesting a more complex molecular mechanism and a non-direct interaction between *SlSTAT* and *SlCBFs*.

### 2.5. The Effect of STAT on RNA m^6^A Modification in Tomato Leaves and Its Relationship with Chilling Resistance

The relationship between SlSTAT and RNA m^6^A modifications in animals, along with the impact of tomato m^6^A modifications on their resistance to cold stress, prompts us to explore the effects of *SlSTAT* expression on the RNA m^6^A modification levels of tomato leaves and the expression of m^6^A-related proteins (writers, readers, and erasers) (Table S6).

**Figure 5:**
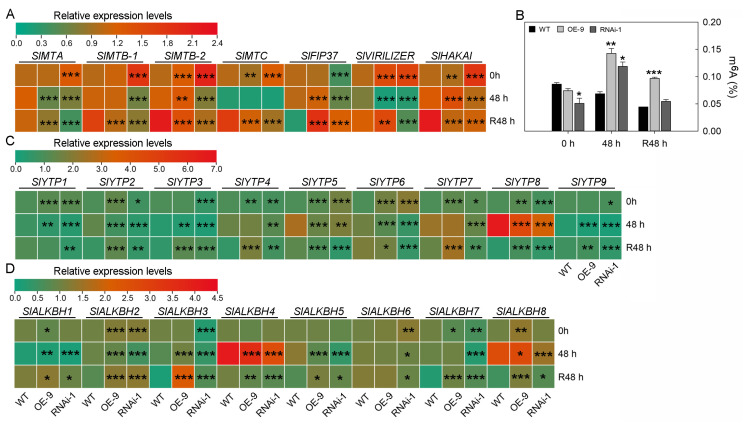
Expressions of *SlSTAT* gene change RNA *N*^6^-methyladenosine (m^6^A) modification levels and m^6^A-related gene expressions under low-temperature stress. (**A**,**C**,**D**) Relative expression levels of m^6^A writer (**A**), reader (**C**), and eraser (**D**) genes in WT and transgenic tomato leaves under low-temperature conditions. The mean expression levels were calculated in comparison to the WT plants at time point 0 h. (**B**) RNA m^6^A modification levels of WT and transgenic tomato leaves under low-temperature conditions. Error bars in the figures represent the SD. Significance analyses were separately conducted at 0 h, 48 h, and R48 h, with all comparisons of significant differences being made against the wild type (WT). Asterisks denote statistical significance levels: *, **, and *** indicate *p* <  0.05, *p* < 0.01, and *p* <  0.001, respectively.

For m^6^A writer proteins, under normal growth conditions, both overexpression and downregulation of *SlSTAT* promote the expression of m^6^A writer subunit genes, except for *SlFIP37*, which was not significantly different in expression between the *SlSTAT* OE plants and the WT plants, and was downregulated in *SlSTAT* RNAi plants (Figure 5A). After 48 h of cold treatment, the expression level of *SlFIP37* in OE plants was higher than in the WT plants, while in the RNAi lines, the expression level was lower than in the WT plants (Figure 5A). At R 48 h, the expression level of *SlFIP37* was higher in both OE plants and RNAi lines compared to the wild type, with the expression level in OE plants being higher than that in the RNAi lines (Figure 5A).

As methyltransferases involved in RNA m^6^A modification, the expression levels of the writer subunit genes reflect, to some extent, the level of m^6^A modification in cellular RNA. As shown in Figure 5B, under normal temperature conditions, the low expression of STAT significantly reduced the m^6^A modification levels in tomato leaves, while the m^6^A modification levels in the OE plant leaves did not show any significant differences compared to WT plants (Figure 5B). After 48 h of low-temperature treatment, the m^6^A modification levels in the leaves of OE plants and RNAi lines were both higher than those in the wild type, with the OE plants showing higher levels than the RNAi lines (Figure 5B). At R 48 h, the RNA m^6^A modification levels in the leaves of OE plants were significantly higher than those in the wild type, whereas the RNA m^6^A modification levels in the leaves of RNAi lines showed no significant difference compared to the wild type (Figure 5B). It is worth noting that, under normal growth condition, the expression level of *SlFIP37* and the RNA m^6^A modification levels in the leaves of both WT and transgenic plants remained consistent (Figure 5A,B). In summary, under normal and low-temperature growth conditions, the expression of *SlSTAT* affects the expression of RNA m^6^A writer genes, ultimately influencing the changes in RNA m^6^A modification levels in tomato leaves.

For m^6^A reader proteins, *SlYTP8* and *SlYTP9* are the two genes that respond most significantly to cold stress. Cold temperatures induce the up-regulation of *SlYTP8*, while *SlYTP9* is downregulated (Figure 5C). Compared to the WT plants, *SlYTP8* and *SlYTP9* exhibited a similar trend in expression level changes in the transgenic plants (Figure 5C). The *SlYTP8*/*9* expression levels in the OE, WT, and RNAi lines decreased sequentially at both 0 h and R48 h of cold treatment, indicating a positive correlation between *SlYTP8/9* expression and *SlSTAT* expression (Figure 5C). However, after 48 h of cold treatment (48 h), the expression of *SlYTP8*/*9* in the *SlSTAT* OE plants was lower than that in the WT (Figure 5C). Nevertheless, the results above indicate that *SlSTAT* may regulate the recognition of m^6^A signals by directly influencing the expression of *SlYTP8* and *SlYTP9* under low-temperature conditions.

For m^6^A eraser proteins, the expression changes of *SlALKBH1*, *SlALKBH4*, and *SlALKBH8* were most pronounced at low temperatures. Low temperatures lead to a downregulation of *SlALKBH1*, while *SlALKBH4* and *SlALKBH8* were up-regulated (Figure 5D). As demethylases involved in m^6^A modification, the expression levels of eraser genes are another important factor influencing the levels of RNA m^6^A modification within cells. Under normal growth conditions, the overexpression of *SlSTAT* resulted in an upregulation of the expression levels of most writer genes; however, the RNA m^6^A modification levels in the OE plants did not show significant differences compared to the WT tomatoes (Figure 5A,B). The reason for the above results may be that the overexpression of *SlSTAT* also led to an upregulation of m^6^A eraser genes. Under normal growth conditions, the eraser gene that showed the most significant upregulation in STAT-overexpressing plants compared to the WT plants was *SlALKBH8* (Figure 5D). In other words, the relatively higher expression level of *SlALKBH8* in the OE plants may explain why, despite the increased expression of m^6^A writer genes in the OE plants, there was no significant difference in m^6^A levels compared to the WT plants (Figure 5A,B,D). Regardless of whether in the WT plants, OE lines, or RNAi lines, the expression of the *SlALKBH8* gene showed a trend of initially increasing and then decreasing during the low-temperature treatment and subsequent recovery to normal growth temperatures (Figure 5D). In addition, the expression level of *SlALKBH8* in the STAT OE lines was higher than in the WT plants at 0 h, 48 h, and R48 h (Figure 5D). Nevertheless, at 0 h, the expression level of *SlALKBH8* in the RNAi lines was not significantly different from that in the WT plants. At 48 h, the expression level of *SlALKBH8* in the RNAi lines was lower than that in the WT plants. At R48 h, the expression level of *SlALKBH8* in the RNAi lines was higher than that in the WT tomatoes (Figure 5D). In summary, *SlSTAT* may regulate the removal of m^6^A signals by directly or indirectly influencing the expression of *SlALKBH8*. Additionally, the differences in expression and interaction of writer and eraser genes in the WT tomatoes, OE lines, or RNAi lines ultimately determine their intracellular RNA m^6^A modification levels, which involve a very complex molecular mechanism.

## 3. Discussion

### 3.1. SlSTAT Family Member Identification and Analysis

The initial STAT protein was discovered in the invertebrate Drosophila, referred to as D-STAT or STAT92E, highlighting its functional significance in invertebrates [23]. Then, Kawata et al. identified a STAT-like protein in *Dictyostelium*, which is also a DNA-binding protein, and its activation is based on an SH2 domain [24]. Subsequently, the STAT signaling pathway was detected in slime molds, nematodes, fruit flies, and vertebrates, while reports of fungi and plant STATs were lacking [25]. There are seven *HsSTAT*s in the human genome, but there is only one SlSTAT in tomato and two AtSTATs in the Arabidopsis genome (Figure S1). We hypothesize that the significant reduction in the number of STATs in plants may be attributed to the following two possible reasons. Firstly, the tomato SlSTAT protein has a high degree of functional enrichment. Secondly, the presence of other proteins in the tomato genome that took on similar functions resulted in the functional degradation of STATs during evolution and ultimately leading to a decrease in their numbers. For example, the phytohormone cytokinin plays diverse roles in plant development and stress response, and it has a similar extracellular-to-nuclear signaling pathway. However, the protein molecules involved in plant cytokinin signaling differ significantly from those in the animal JAK–STAT pathway [26]. Fleishon et al. have investigated the cross-talk between gibberellin and cytokinin in tomato (*S. lycopersicum*) [27]. Whether the function of tomato STAT proteins exhibits functional redundancy or can be effectively compensated by other regulatory proteins requires systematic investigation to clarify the molecular mechanisms underlying STAT-mediated signaling transduction.

As Figure 1C shows, the SlSTAT protein is localized in the nucleus and cytoderm. It has been confirmed in animals that the subcellular localization of STAT is related to its phosphorylation [25]. When STAT is unphosphorylated, it resides in the cytoplasm. Upon the binding of cytokines to membrane receptors, JAK is recruited and phosphorylated, which then recruits STAT, causing STAT to aggregate at the receptor. Then, upon phosphorylation, STAT proteins translocate to the cell nucleus, where they undergo conformational changes that facilitate their binding to specific DNA sequences and initiate gene transcription [5,25]. Consequently, STAT proteins can be detectable in both the cytoplasm and the nucleus, corroborating the findings of this study.

### 3.2. Expression Levels of SlSTAT in Different Tomato Tissues and Its Function in Seed Germination

In animals, the STAT protein family comprises numerous members whose amino acid sequence diversity, coupled with tissue-specific expression patterns, offers substantial variability to account for their various functions in mediating responses to extracellular signaling proteins [3,5,25]. For example, the absence of STAT and N-Myc disrupts normal mammary cell development and elevates the metastatic potential of mammary tumor cells [28]. Despite the presence of a singular *STAT* gene in the tomato genome, *SlSTAT* demonstrates distinct expression patterns across different developmental stages and in different tissue/organs (Table S4; Figure 2).

Seed germination is a critical stage in the plant life cycle, and it plays an important role in the efficiency of agricultural production [16]. The increased expression from the early germination period to the seedling growth stage underscores the critical role of *SlSTAT* in the seed germination process (Table S4; Figure 2). Further, the expression of the *SlSTAT* gene in tomatoes affects seed germination, particularly at low expression levels. After 3 and 8 days of germination, the low expression of *SlSTAT* significantly reduced the germination rate and the fresh weight of seedlings, respectively, thereby delaying the germination process (Figure 3). These experimental results highlight the important role of STAT in seed germination. Nevertheless, increasing the expression level of tomato *SlSTAT* did not have a significant impact on germination rate and biomass (Figure 3). Based on this, it is speculated that normal tomato growth does not require a high level of *SlSTAT* gene expression, as there is a cascading amplification effect in the signaling transduction process. Additionally, the overall expression level of *SlSTAT* is relatively low during different tomato growth periods and in various tissue/organs (Table S4; Figure 2), which further support our speculation.

### 3.3. Expression of SlSTAT Changes the Chilling Resistance of Tomato

In addition to tomato growth and development, roles of *SlSTAT* in stress responses were examined, alongside initial investigations into its potential molecular mechanisms. *SlSTAT* responds to low-temperature treatment and shows a downregulation expression trend (Figure 4A). Moreover, the overexpression and downexpression of *SlSTAT* enhanced tomato cold stress resistance and sensitivity, respectively, by affecting peroxide accumulation in the leaves and the activity of enzymes associated with reactive oxygen species scavenging (Figure 4B–G).

When it comes to their potential molecular mechanisms, STAT proteins could bind to specific DNA sequences in the nuclear and initiate gene transcription [5]. There are various identified DNA-binding proteins (transcription factors, TFs) that modulate the expression of downstream stress-responsive genes either positively or negatively in the cold signaling network of tomato [18]. Among these, the DREB1/CBF TFs are pivotal regulators within the plant’s cold response transcriptional regulation network [29] (Zhao et al., 2015). In Arabidopsis and tomato, these *CBF* genes are rapidly activated under cold stress, initiating a transcriptional cascade that results in the expression of a wide array of cold-induced genes [30,31,32]. This inevitably leads us to consider whether there are connections between *STAT* and *CBF*s. Three *CBF*s exist in the tomato genome: *SlCBF1*, *SlCBF2*, and *SlCBF3* [32]. Interestingly, both overexpression and underexpression of *SlSTAT* not only influence the expression of *SlCBF* genes under normal growth conditions but also affect the expression of these three genes after low-temperature treatment and during the growth recovery growth, particularly at R48 h (Figure 4H–J). However, the changes in the expression of *SlCBF1*-*3* in transgenic plants do not exhibit a simple linear relationship with the variations in *SlSTAT* expression, indicating a more complex molecular mechanism and a non-direct interaction between *STAT* and *CBF*s. Nevertheless, the experimental results still suggest an interaction between the STAT signaling pathway and DREB1/CBF TF-centered transcriptional regulatory network in tomato cold stress response, which provide directions for further in-depth research in tomatoes and other plants.

### 3.4. The Effect of SlSTAT on RNA m^6^A Modification in Tomato Low-Temperature Stress Response

Furthermore, the essential roles of RNA m^6^A modification in plant low-temperature stress response and reports on the relationship between STAT and m^6^A modification in animals prompted us to identify the effect of *SlSTAT* on RNA m^6^A modification in tomato low-temperature stress response [33,34,35]. RNA m^6^A modification is one of the most common RNA modification types in eukaryotes, which is catalyzed by m^6^A writer (methyltransferase), bound and recognized by m^6^A reader, and removed by m^6^A eraser (demethylases) proteins [11,12]. The influence of *SlSTAT* on the RNA m^6^A modification can be examined from four perspectives: its effects on m^6^A writer genes, reader genes, eraser genes, and the overall levels of RNA m^6^A modification.

As shown Figure 5A,B, both the upregulation and downregulation of the *SlSTAT* gene affect the expression of m^6^A writer genes, no matter whether under normal or low-temperature growth conditions (Figure 5A). The aforementioned results primarily demonstrate the impact of STAT on the expression of m^6^A writer genes. In contrast, the literature on animals has reported the influence of m^6^A modification on the expression of *STAT*. In porcine bone marrow-derived stem cells (pBMSCs), knockout of the m^6^A “writer” protein methyltransferase-like 3 (METTL3) resulted in the reduced m^6^A methylation of JAK mRNAs, which in turn increases JAK protein levels. This enhancement promotes adipogenesis via the JAK1/STAT5/C/EBPβ pathway in pBMSCs adipogenic differentiation, highlighting a coordinated network that connects RNA m^6^A methylation to the JAK–STAT signaling pathway [36]. In tumor-infiltrating myeloid cells (TIMs), METTL3-mediated m^6^A modification of Jak1 mRNA enhances JAK1 protein translation efficiency through the m^6^A-YTHDF1 axis, leading to increased phosphorylation of STAT3 [37]. Moreover, existing literature documents direct interactions between STAT and m^6^A writer complex in animals. The interaction between METTL3 (m^6^A writer) and STAT5B promotes co-transcriptional m^6^A modification of mRNA, thereby enhancing breast tumorigenesis [10]. Therefore, the relationship between *STAT* and m^6^A writer gene expressions is not unidirectional.

For m^6^A reader proteins, *SlYTP8* and *SlYTP9* are the two genes that responded most significantly to cold stress (Figure 5C) [38,39]. Moreover, the transgenic tomatoes overexpressing the *SlYTP8* gene exhibited less resistance to chilling stresses compared to WT plants [38]. The *SlYTP8* expression levels in the *SlSTAT* OE, WT, and *SlSTAT* RNAi lines decreased sequentially at both 0 h and R48 h of cold treatment, indicating a positive correlation between *SlYTP8* and *SlSTAT* expressions (Figure 5C). It is possible that *SlSTAT* enhances the expression of *SlYTP8*, increasing its recognition of m^6^A signals. Nevertheless, the existing reports only provide evidence that reader proteins influence STAT signaling transduction. YTHDF2 (an m^6^A reader) facilitates the degradation of STAT1 mRNA by recognizing its m^6^A modifications, thereby influencing glycolysis and the polarization of M1 macrophages [40]. Additionally, in RNA virus-infected cells, YTHDF3 has a positive role in antiviral JAK–STAT signaling [41].

From the m^6^A eraser perspective, the expression changes of *SlALKBH1*, *SlALKBH4*, and *SlALKBH8* are most pronounced at low temperatures (Figure 5D). Shen et al. also reported that *SlALKBH8* (*Solyc12g096230.2*) was upregulated at 24 and 48 h of low-temperature treatment [39]. Moreover, considering the positive correlation between the expression of *SlSTAT* and *SlALKBH8, SlSTAT* may regulate the removal of m^6^A signals by directly or indirectly influencing the expression of *SlALKBH8* (Figure 5D). On the other hand, the m^6^A eraser could also alter the signaling transduction of the JAK–STAT pathway. FTO (m^6^A eraser) increased the stability of SOCS gene mRNA and thus negatively regulated the JAK–STAT signaling pathway [42]. Conversely, FTO plays a positive role in bladder cancer by maintaining STAT3 mRNA stability through decreasing its m^6^A modification levels [13].

The differences in expression and interaction between writer and eraser genes in the WT tomatoes, *SlSTAT* OE lines, or RNAi lines ultimately determine their RNA m^6^A modification levels, which involve a very complex molecular mechanism (Figure 5B). Although there are no reported studies on the impact of STAT on the overall intracellular RNA m^6^A modification levels, the research results of Yang et al. and Wang et al. have fully demonstrated the important regulatory role of m^6^A modification in tomato’s response network to low-temperature stress [35,43]. The aforementioned research provides a foundational basis for exploring how the STAT-m^6^A axis participates in tomato low-temperature resistance.

In summary, unlike existing literature, which focuses more on the effects of m^6^A modifications on STAT/JAK–STAT signal transduction, this study preliminarily analyzed the potential molecular mechanisms by which STAT influences m^6^A modifications in relation to tomato cold stress resistance. This provides a new perspective for exploring the interactions between the JAK–STAT and m^6^A modification signaling pathways.

## 4. Materials and Methods

### 4.1. Identification and Analysis of the SlSTAT Gene Family

Firstly, protein sequences of 7 human (*Homo sapiens*) STATs (HsSTAT1-4,5A-B,6) were downloaded from the NCBI website (https://www.ncbi.nlm.nih.gov/; accessed on 30 March 2025). Then, BLASTP analysis was conducted via TBtools (version 2.136) using these human STAT sequences to identify STAT family members in Arabidopsis (*Arabidopsis thaliana*) and tomato (*S. lycopersicum*) [44]. During this process, Arabidposis genome files were download from TAIR (https://www.arabidopsis.org/; accessed on 30 March 2025), and tomato genomes were obtained from Sol Genomics Network (https://solgenomics.net/; accessed on 30 March 2025). In addition, a phylogenetic tree showing the evolutionary relationships among humans, Arabidopsis, and tomato, was constructed using MEGA7.0 software, with parameters set to 1000 bootstrap replicates [38].

### 4.2. Gene Clone and Gene Structure Analysis

A total of 30 DPG (30 Days Past Germination) Micro-tom leaves were used as plant materials for total RNA isolation. The FlashPure Plant Total RNA Mini Kit (R019, GeneBetter life science, Beijing, China, http://www.gene-better.cn/; accessed on 30 March 2025) and RevertAid First Strand cDNA Synthesis Kit (K1622, Thermo Scientific, Shanghai, China, https://www.thermofisher.cn/; accessed on 30 March 2025) were used to conduct total RNA extraction and synthesis of first-strand complementary DNA (cDNA) experiments, respectively, according to manufacturer’s guidelines. Table S7 presents the primers for gene cloning, which were designed using Primer Premier 6.0. The coding sequence (CDS) of SlSTAT was obtained through PCR (polymerase chain reaction) amplification using the aforementioned cDNA and gene cloning primers. The genome sequence of *SlSTAT* was also download form Sol Genomics Network (https://solgenomics.net/; accessed on 30 March 2025). Then, gene structure analysis was conducted using TBtools, comparing CDS and the genome sequence of *SlSTAT* [44].

### 4.3. Promoter Clone and Cis-Acting Element Analysis

A total of 30 DPG Micro-tom roots were used as plant materials for the genomic DNA isolation experiments. Then, referring to the instructions of the Hi-Fast Plant Genomic DNA Kit (D200, GeneBetter life science, Beijing, China, http://www.gene-better.cn/; accessed on 30 March 2025), the DNA of these tomato roots was isolated. Promoter clone PCR primers were designed based on about 1500 bp of the gene’s upstream sequence, which was downloaded from the tomato genome available on Sol Genomics Network (https://solgenomics.net/; accessed on 30 March 2025) (Table S7). For promoter *cis-*acting element predictions, Lescot et al. provides the useful PlantCARE online prediction tool [45].

### 4.4. Sub-Cellular Localization

pCambia2301, as a plant overexpression vector, contains a green fluorescent protein (GFP) label. *SlSTAT* CDSs were inserted into this vector after being PCR amplified (see vector construction primers in Table S7). Positive recombinant plasmids, pCambia2301-*SlSTAT*, were transferred to onion epidermal cells with the help of *Agrobacterium tumefaciens* (strain GV3101). After infection and growth for 48 h at 22 °C, the onion samples were placed onto glass slides and examined under a fluorescence microscope (Olympus; Tokyo, Japan) equipped with a 20× objective lens.

### 4.5. Plot Cartoon Heatmap

Cartoon heatmap construction methods were described by Li et al. [22]. Briefly, schematic diagrams of tomato tissues/organs, gene expression values (FPKM; Fragments Per Kilobase of transcript per Million mapped reads), and the *SlSTAT* gene ID were imported into TBtools, then the cartoon heatmap could be downloaded from the pop-up page [22,44]. Among them, schematic diagrams of tomato tissues/organs and their gene expression values were both provided by the tomato transcription database established by Li et al. [22].

### 4.6. Plant Growth and Treatments

The seeds of Micro-Tom utilized in this study are those stored in our laboratory (College of Life Sciences and Medicine, Shandong University of Technology, Zibo, Shandong, China). Micro-Tom seedlings were cultivated in plastic pots (7 × 7 cm) containing a mixture of soil, perlite, and vermiculite (4:1:1, *v*:*v*:*v*) as the growth substrate. The light intensity was maintained at 5000 lux, with a day/night cycle of 16 h of light followed by 8 h of darkness. The temperature regimen during the day and night periods was set at 25 °C and 20 °C, respectively.

For the seed germination experiments, seeds of wild-type (WT) and transgenic tomato plants were then put on 1/2 MS medium after surface disinfection with 70% ethanol (*v*:*v*) for 1 min, 5% sodium hypochlorite for 10 min, and sterile water for 3–5 times. The light intensity was maintained at 2000 lux, with a day/night cycle of 14 h of light followed by 10 h of darkness. The temperature regimen during the day and night periods was set at 25 °C and 20 °C, respectively. Phenotype analysis was conducted at 0, 3, and 8 d, and at least five biological replicates were used.

For low-temperature (4 °C) treatment experiments, 30 DPG tomato seedlings of WT plants were used as experimental materials. While keeping the light intensity and photoperiod constant, the temperature inside the growth chamber was adjusted to 4 °C. Leaf samples for *SlSTAT* qRT-PCR (quantitative reverse transcription-PCR) analysis were collected at 0, 1, 3, 6, 12, 24, 48 h, and after a recovery period of 48 h (R48 h) [46].

For the low-temperature (4 °C) comparison experiments, 30 DPG tomato seedlings of both WT and transgenic plants were used as experiment materials, and the treatment methods were the same as those used in the aforementioned low-temperature (4 °C) treatment experiments. For physiological index assessment, leaf samples collected at 0 h, 48 h, and R48 h were utilized. The time point selection for measuring physiological indicators was based on the expression pattern of *SlSTAT* under low-temperature treatment conditions. Moreover, the leaf samples for *SlCBFs* and m^6^A-related gene qRT-PCR analysis were also collected at 0 h, 48 h, and R48 h.

### 4.7. qRT-PCR

For qRT-PCR, cDNA synthesis was conducted according to the manuscript instruction of the PrimeScript™ RT Reagent Kit with gDNA Eraser (Perfect Real Time) (RR047A, Takara, Beijing, China, https://www.takarabiomed.com.cn/; accessed on 30 March 2025). Then, qRT-PCR was conducted with TB Green^®^ *Premix Ex Taq*™ II FAST qPCR (CN830A, Takara, Beijing, China, https://www.takarabiomed.com.cn/; accessed on 30 March 2025) via the LightCycler 96/LightCycler 480 System (Roche Diagnostics; Indianapolis, IN, USA). Three technical replicates were used for each cDNA temple. *SlACTIN (Solyc11goo5330*) was used as the reference gene [19]. qRT-PCR gene-specific primer sequences of *SlACTIN* and m^6^A modification-related genes are shown in Table S7 and were download from Shen et al., respectively [39].

### 4.8. Tomato Transformation and Identification

pCambia2301-*SlSTAT* obtained at Section 4.4 can also be used as plant OE (overexpression) vectors. For RNAi (RNA interference) plasmid construction, 200-bp specific sequence of *SlSTAT* CDS was inserted into pK7WWG2D plasmids according to the manuscript instruction of the Gateway™ BP Clonase™ II Enzyme Mix and (11789020, Invitrogen, Thermo Fisher Scientific, Shanghai, China, https://www.thermofisher.cn/; accessed on 30 March 2025) and Gateway™ LR Clonase™ II Enzyme Mix (11791019, Invitrogen, Thermo Fisher Scientific, Shanghai, China, https://www.thermofisher.cn/; accessed on 30 March 2025). The primers used for RNAi plasmid construction are listed in Table S7. The *SlSTAT*-200 fragment, cloned using the aforementioned primers, was then ligated into the pDONR vector using Gateway™ BP Clonase™ II Enzyme at 25 °C for 1 h. The resulting pDONR-SlYTP9-200 was mixed with the pK7GWIWG2D vector and incubated with Gateway™ LR Clonase™ II Enzyme at 25 °C for 1 h, ultimately yielding pK7WWG2D-*SlSTAT*-200. The Gateway™ BP Clonase™ II Enzyme and Gateway™ LR Clonase™ II Enzyme are provided in the kits, while the empty pDONR and pK7WWG2D vectors are those maintained in the laboratory (College of Life Sciences and Medicine, Shandong University of Technology, Zibo, Shandong, China). Recombinant plasmids (pCambia2301-*SlSTAT* and pK7WWG2D-*SlSTAT*-200) were applied to the tomato transformation process.

The GV3101 strain containing either the pCambia2301-*SlSTAT* or pK7WWG2D-*SlSTAT*-200 constructs was employed to infect Micro-Tom tomato cotyledons at Wuhan Biorun Biotechnology Co., Ltd., Wuhan, China. The selection of putative transgenic plants was conducted using Kanamycin at a concentration of 50 mg·L^−1^, followed by confirmation at the DNA level using GUS gene primers for OE lines and GFP gene primers for RNA RNAi lines (see Table S7). Transgenic lines exhibiting a segregation ratio of 3:1 were selected, and homozygous T_3_ generation transgenic lines were further verified using qRT-PCR methods.

### 4.9. Measurement of Stress-Related Physiological Indexes

The concentrations of malondialdehyde (MDA) and hydrogen peroxide (H_2_O_2_), as well as the activities of superoxide dismutase (SOD), catalase (CAT), and peroxidase (POD), were assessed according to established protocols using the following test kits: Malondialdehyde Test Kit (MDA-2-Y, Comin, Suzhou, China, http://www.cominbio.com/index.html; accessed on 30 March 2025), Hydrogen Peroxide Test Kit (H2O2-2-Y, Comin), Superoxide Dismutase Test Kit (SOD-2-Y, Comin), Catalase Test Kit (CAT-2-W, Comin), and Peroxidase Test Kit (POD-2-Y, Comin). Detailed experimental procedures are referenced in Zhang et al. [38]. Three technical replicates were used for each sample.

### 4.10. RNA m^6^A Modification Level Identification

Following total RNA extraction, the m^6^A methylation level was quantified utilizing the EpiQuik m^6^A RNA Methylation Quantification Kit (EPIGENTEK, Farmingdale, NY, USA; https://www.epigentek.com; accessed on 30 March 2025), in accordance with the methodology outlined by Hu et al. [47]. In summary, 200 ng of total RNA was immobilized on strip wells coated with m^6^A-specific antibodies. Following a wash step, the detection and capture antibody solutions were introduced, and the signal was quantified by measuring absorbance at 450 nm. The m^6^A levels in the samples were determined using a standard curve. The negative control (NC) comprised RNA devoid of m^6^A, while the positive control (PC) was an m^6^A oligonucleotide calibrated to contain 100% m^6^A, both supplied by the kit. According to the kit instructions, two technical replicates were used for each RNA sample.

### 4.11. Statistical Analysis

Statistical analysis was conducted using GraphPad Prism (version 8.0.1; GraphPad, La Jolla, CA, USA). Variables between two independent groups were compared using the unpaired *t*-test (two-tailed). A significance level of *p* < 0.05 was considered indicative of statistical significance.

## 5. Conclusions

SlSTAT is the only STAT protein found in the tomato genome, and it is localized in both the nucleus and the cytoplasm. In terms of tomato growth and development, the low expression of *SlSTAT* significantly reduced seed germination rate and seedling fresh weight, thereby delaying the germination process. Regarding tomato stress responses, overexpression and downexpression of *SlSTAT* enhanced cold stress resistance and sensitivity, respectively. The changes in cold resistance may be achieved by influencing the gene expression of *SlCBF*s or through RNA m^6^A modifications. Although the above findings fill gaps in the research on tomato and plant STAT proteins, the specific functions of SlSTAT in tomato seed germination and cold stress responses, as well as the interactions between the JAK–STAT signaling pathway and m^6^A modifications, still require further in-depth exploration.

## Data Availability

The original contributions presented in this study are included in the article/Supplementary Material. Further inquiries can be directed to the corresponding author.

## Supplementary Materials

The supporting information can be downloaded at: https://www.mdpi.com/article/10.3390/ijms26073338/s1.

## Abbreviations

Catalase (CAT); coding sequence (CDS); cDNA (complementary DNA); days past germination (DPG); genomic DNA (gDNA); Fragments Per Kilobase of transcript per Million mapped reads (FPKM); green fluorescent protein (GFP); hydrogen peroxide (H_2_O_2_); interferons (IFNs); Janus kinase–signal transducer and activator of transcription (JAK-STAT); malondialdehyde (MDA); messenger RNA (mRNA); negative control (NC); no significance (ns); *N*^6^-methyladenosine (m^6^A); overexpression (OE); peroxidase (POD); polymerase chain reaction (PCR); positive control (PC); qRT-PCR (quantitative reverse transcription polymerase chain reaction); RNA interference (RNAi); superoxide dismutase (SOD); Standard Deviation (SD); untranslated regions (UTRs); wild-type (WT).
