# Supplementary material for: Functions of Tomato (Solanum lycopersicum L.) Signal Transducer and Activator of Transcription (STAT) in Seed Germination and Low-Temperature Stress Response
Source: Int J Mol Sci. 2025 Apr 3;26(7):3338. doi: 10.3390/ijms26073338 (PMC11989334; doi:10.3390/ijms26073338)
Supplement: Supplementary file 1 [file ijms-26-03338-s001.zip › Supplementary Figure S1-S2.pdf]

Figure S1

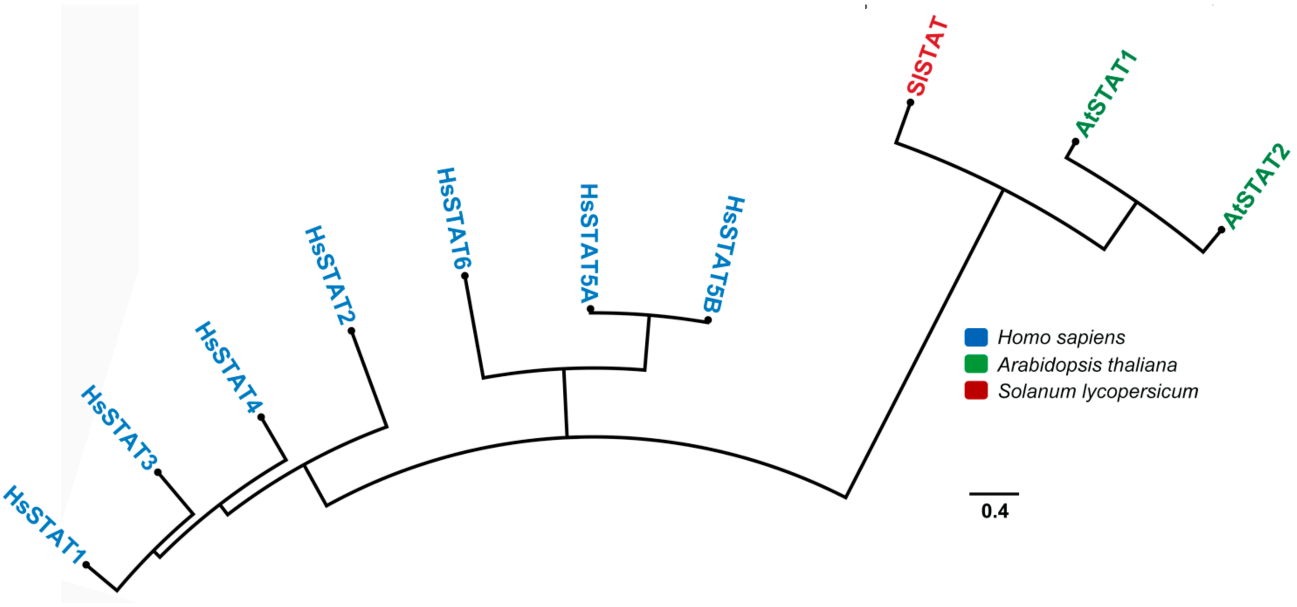

Figure S1. Unrooted phylogenetic tree of STATs from *Homo Sapiens*, *Arabidopsis thatliana*, and *Solanum lycopersicum*. The phylogenetic tree was constructed by the neighbor-joining method using MEGA7 software with 1000 bootstrap replicates. The scale bar corresponds to a 0.4 genetic distance.

Figure S2

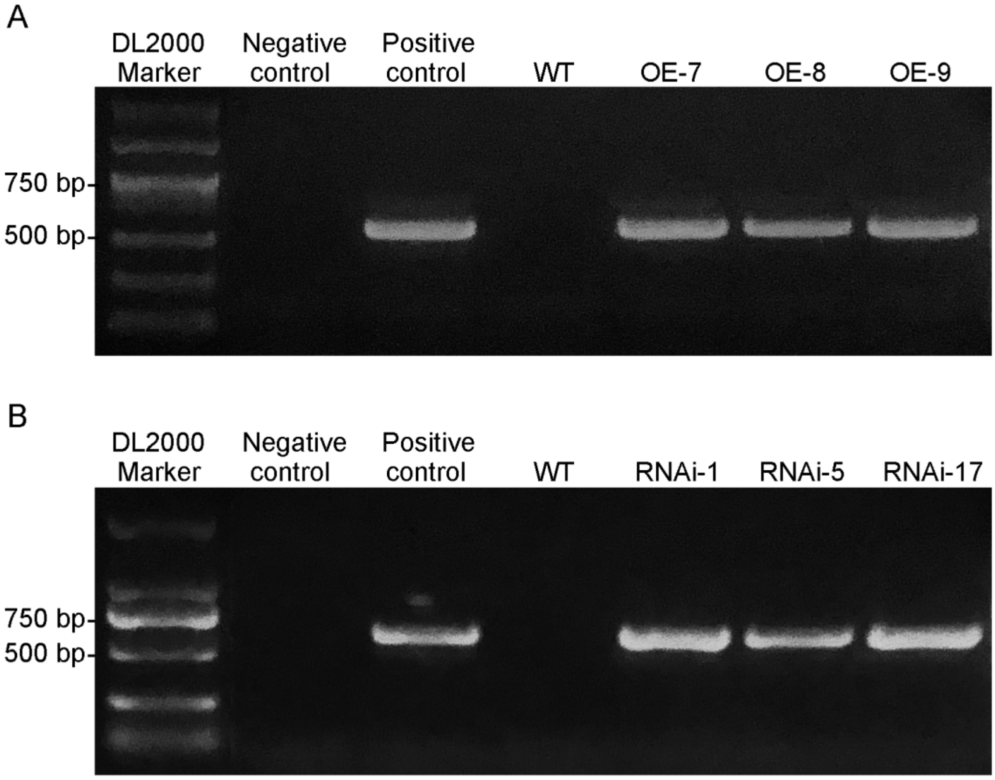

Figure S2. DNA-level identification of *SlSTAT* OE and RNAi transgenic plants. **(A)** Results of PCR electrophoresis, using DNA of WT or STAT OE lines (OE-7, OE-8, and OE-9) as the PCR temples. *GUS* gene primers (Table S1) are designed according to the *GUS* gene sequence in the OE vector, pCambia2301. **(B)** Results of PCR electrophoresis, using DNA of WT or STAT RNAi lines (RNAi-1, RNAi-5, and RNAi-17) as the PCR temples. e*GFP* gene primers (Table S1) are designed according to the *eGFP* gene sequence in the RNAi vector, pK7WWG2D. Negative control means the PCR temple is double steamed water. Empty pCambia2301 and pK7WWG2D vector were used as PCR temple in positive controls of OE and RNAi transgenic plants, respectively.
